# Supplementary material for: Bifidobacterium longum subsp. infantis CECT 7210 Reduces Inflammatory Cytokine Secretion in Caco-2 Cells Cultured in the Presence of Escherichia coli CECT 515
Source: Int J Mol Sci. 2022 Sep 16;23(18):10813. doi: 10.3390/ijms231810813 (PMC9503999; doi:10.3390/ijms231810813)
Supplement: Supplementary file 1 [file ijms-23-10813-s001.zip › ijms-1872445-supplementary.pdf]

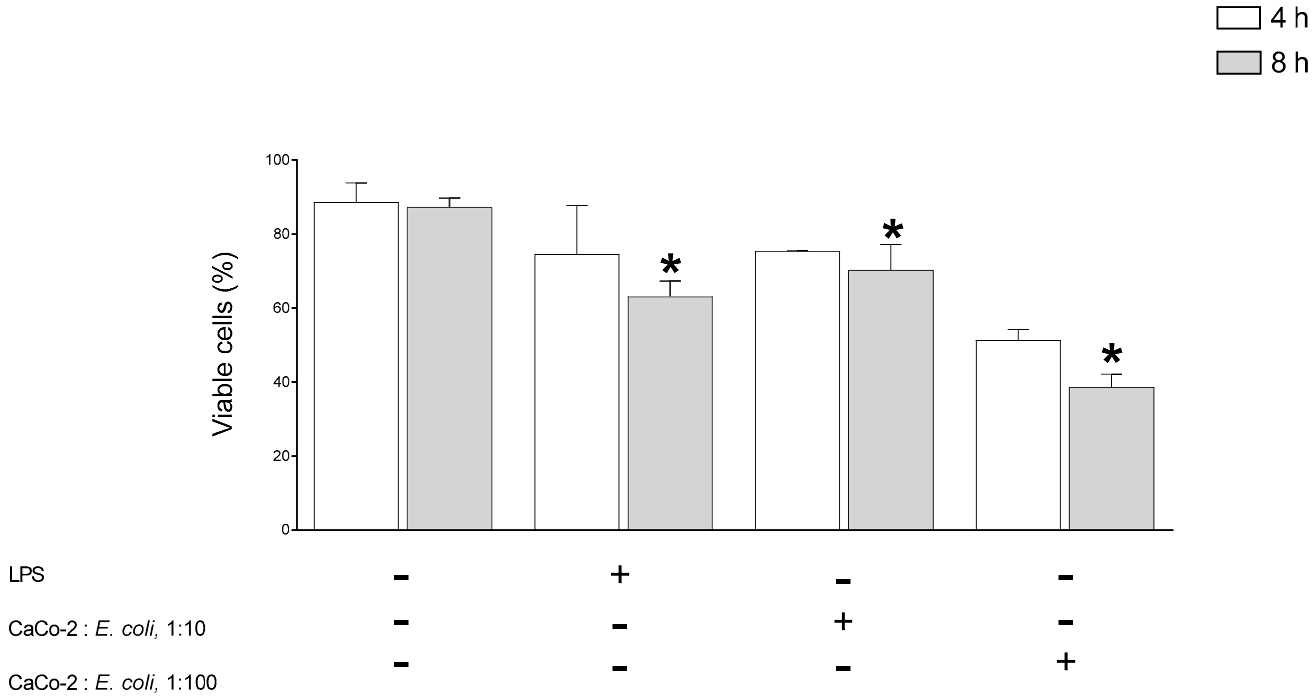

Supplementary Figure S1. Flow cytometry analysis of Caco-2 cell viability. Incubation of Caco-2 cells in the presence of LPS or *E. coli* CECT 515 (ratios 1:10 and 1:100, Caco-2:*E. coli*) for 4 (white bars) and 8 hours (grey bars. Results are expressed in percentages as mean ± SEM of three independent experiments. *p < 0.05 vs. control (untreated cells).

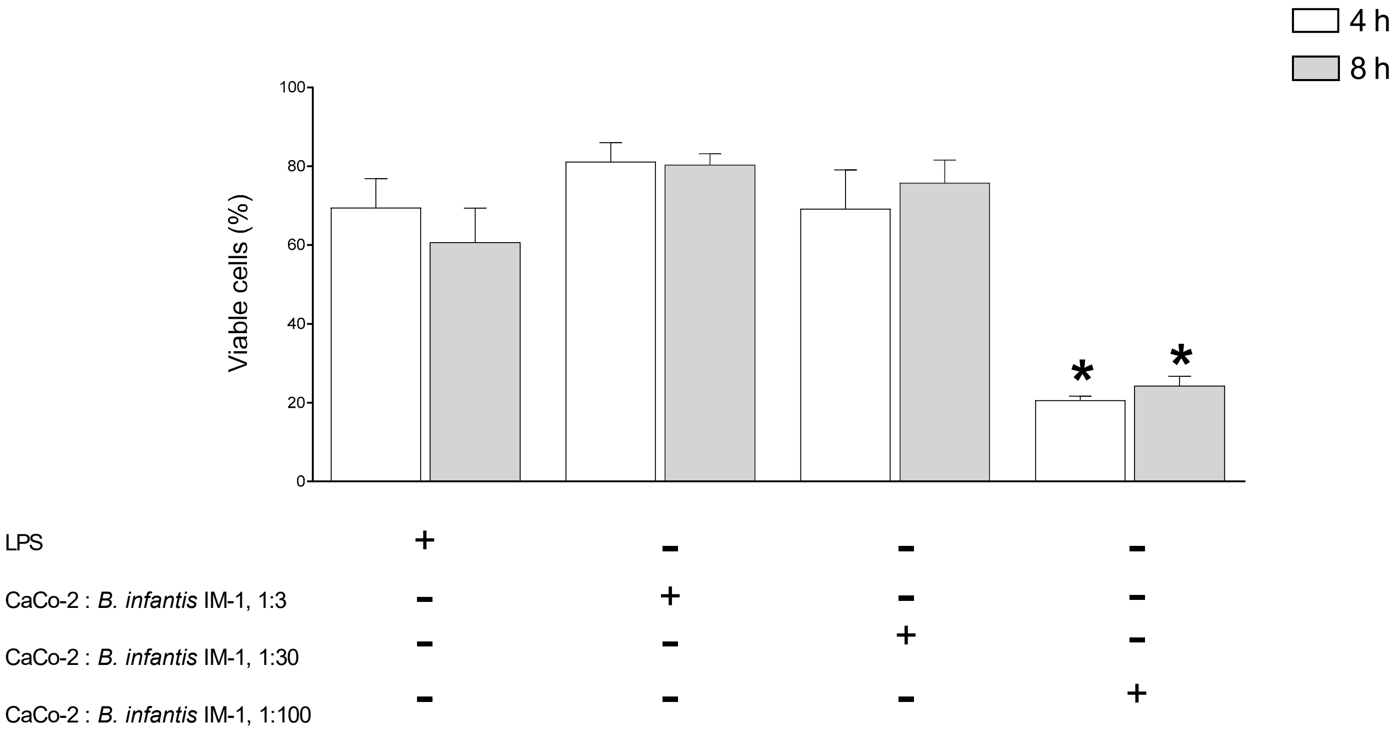

Supplementary Figure S2. Flow cytometry analysis of Caco-2 cell viability. Incubation of Caco-2 cells in the presence of *B. infantis* IM-1® (ratios 1:3, 1:30 and 1:100, Caco-2:*B. infantis* IM-1®) or *B. infantis* IM-1® for 4 (white bars) and 8 hours (grey bars). Results are expressed in percentages as mean ± SEM of three independent experiments. *$p < 0.05$ vs. control (untreated cells).
